# Oxidative Stress, Glutaredoxins, and Their Therapeutic Potential in Posterior Capsular Opacification

**DOI:** 10.3390/antiox13101210

**Published:** 2024-10-08

**Authors:** Chenshuang Li, Weijia Yan, Hong Yan

**Affiliations:** 1 Shaanxi Eye Hospital, Xi’an People’s Hospital (Xi’an Fourth Hospital), Affiliated People’s Hospital of Northwest University, Xi’an 710004, China; lichenshuang0926@163.com; 2 Eye Center, The Second Affiliated Hospital, School of Medicine, Zhejiang University, Zhejiang University Eye Hospital, Hangzhou 310009, China; yanweijia1994@163.com; 3 Institute of Medical Research, Northwestern Polytechnical University, Xi’an 710072, China

**Keywords:** posterior capsular opacification, epidemiology, pathogenesis, oxidative stress, glutaredoxins, advanced antioxidative pharmacological agent

## Abstract

Posterior capsular opacification (PCO) is the most common long-term complication of cataract surgery. Traditionally, the pathogenesis of PCO involves the residual lens epithelial cells (LECs), which undergo transdifferentiation into a myofibroblast phenotype, hyperproliferation, matrix contraction, and matrix deposition. This process is driven by the marked upregulation of inflammatory and growth factors post-surgery. Recently, research on the role of redox environments has gained considerable attention. LECs, which are in direct contact with the aqueous humour after cataract surgery, are subjected to oxidative stress due to decreased levels of reduced glutathione and increased oxygen content compared to contact with the outer fibre layer of the lens before surgery. In this review, we examine the critical role of oxidative stress in PCO formation. We also focus on glutaredoxins (Grxs), which are antioxidative enzymes produced via deglutathionylation, their protective role against PCO formation, and their therapeutic potential. Furthermore, we discuss the latest advancements in PCO therapy, particularly the development of advanced antioxidative pharmacological agents, and emphasise the importance and approaches of anti-inflammatory and antioxidant treatments in PCO management. In conclusion, this review highlights the significant roles of oxidative stress in PCO, the protective effects of Grxs against PCO formation, and the potential of anti-inflammatory and antioxidant therapies in treating PCO.

## 1. Introduction

The most common cause of blindness worldwide is lens opacification, which prevents light from passing through the eye [1]. Cataracts are responsible for 17.01 million cases of blindness, constituting about half of all blindness cases, and account for 83.48 million cases of moderate to severe vision impairment [2,3]. Currently, surgery is the only available treatment for cataracts. The lens comprises lens fibres and a lens capsule. Cataract surgery involves continuous anterior curvilinear capsulorhexis, removing the lens fibres and placing an intraocular lens (IOL) in the capsular bag to ensure proper refraction and clear vision. However, cataract surgery cannot completely eliminate the lens epithelial cells (LECs) in the capsular bag. Residual LECs in the anterior and equatorial capsules can migrate and proliferate onto the posterior capsule, leading to capsular shrinkage, turbidity, and posterior capsule opacification (PCO), which adversely affects vision [4,5].

**Figure 1:**
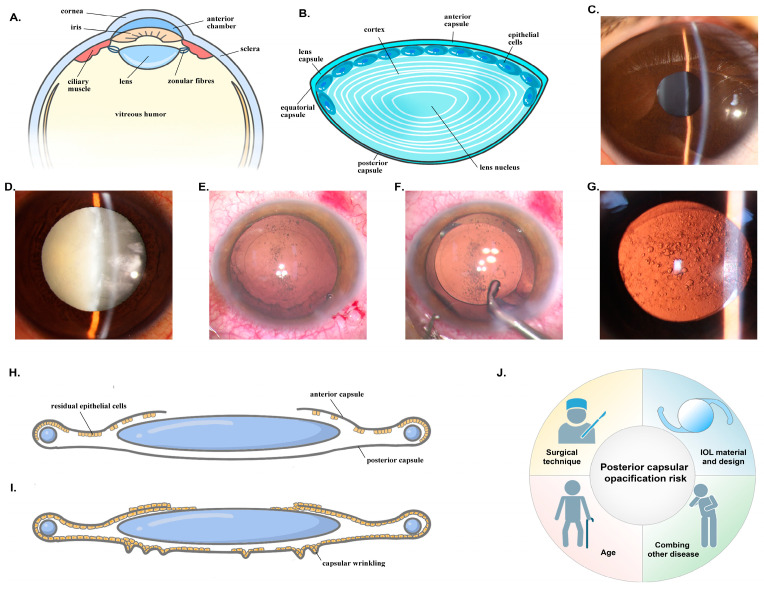
The structure of the lens and formation of PCO. (**A**) Position of the lens; (**B**) Structure of the lens; (**C**) Anterior segment photograph of a normal person; (**D**) Anterior segment photograph of a cataract patient; (**E**) Intraoperative photograph of capsular bag; (**F**) Intraoperative photograph of placement of the intraocular lens; (**G**) Photograph of a patient with PCO; (**H**) Structure of capsular bag and intraocular lens after cataract surgery; (**I**) Structure of capsular bag and intraocular lens in the development of PCO; (**J**) Risk factors of PCO.

PCO is the most common long-term complication following cataract surgery. It can be classified into two types: fibrotic PCO and regenerative PCO. Fibrotic PCO involves fibrotic processes in LECs, such as hyperproliferation, matrix contraction, matrix deposition, and epithelial cell transdifferentiation into a myofibroblast phenotype. Regenerative PCO is characterised by lens fibre cell differentiation and the formation of Soemmerring’s ring and Elschnig’s pearls [4]. Soemmerring’s rings consist of a ring of LEC-derived cells that grow along the periphery of an aphakic lens capsule or around an intraocular lens. Elschnig’s pearls are LEC-derived cells gathering in clusters, resembling pearls in the posterior capsule. The incidence of PCO ranges from 4.7% to 18.6% at three years post-surgery and from 7.1% to 22.6% at five years [6]. Risk factors for PCO include age, the presence of other diseases, IOL material and design, and surgical techniques [7]. In paediatric cataract surgery, the incidence of PCO approaches 100% due to the high regenerative and proliferative capacities of paediatric LECs [8]. Conditions such as diabetes mellitus [9], highly myopic [10], uveitis [11,12], and glaucoma [13] also increase the risk of PCO. Round-edged IOLs are more likely to cause PCO than sharp-edged IOLs [14], and hydrophilic IOL materials are additional risk factors [15]. Although complete removal of LECs is challenging, time-consuming, and increases surgical stress, theoretically, PCO could be prevented by polishing the posterior capsule and eliminating residual LECs [16] (Figure 1).

Neodymium-doped yttrium aluminium garnet (Nd:YAG) laser capsulotomy is commonly used to treat PCO. However, it imposes an economic burden on patients and healthcare systems and has adverse effects, including increased intraocular pressure, iritis or uveitis, and cystoid macular oedema [17]. Additionally, it can cause IOL movement, refractive changes, and IOL damage or pitting [17,18]. With advancements in cataract surgery techniques and the widespread application of functional IOLs, modern cataract surgery has gradually evolved from vision restoration to refractive surgery. The long-term stable positioning of functional IOLs is essential for maintaining their intended function, but PCO and Nd:YAG capsulotomy can compromise this stability. Therefore, it is crucial to understand the mechanisms leading to PCO development and to explore preventive and therapeutic measures.

## 2. Pathogenesis

### 2.1. Cytokines

Following cataract surgery, mechanical injury and disruption of the blood–aqueous barrier result in a marked upregulation of inflammatory and growth factors. These factors are crucial for the proliferation, migration, and transdifferentiation of LECs [4]. Research indicates that transforming growth factor β (TGF-β) promotes epithelial–mesenchymal transition (EMT) in LECs and differentiates into fibroblasts and lens fibre cells in PCO via both Smad-dependent [19,20] and Smad-independent pathways. The latter includes signalling pathways such as mitogen-activated protein kinase/extracellular regulated protein kinase 1/2 (ERK) [21] and RhoA/Rho-associated, coiled coil-containing protein kinase [22]. Additionally, growth factors like fibroblast growth factor (FGF) [23,24], epidermal growth factor (EGF) [25,26], vascular endothelial growth factor (VEGF) [27], interleukins [28,29], and thrombin [30] contribute to LEC survival, proliferation, migration, and the increased synthesis and deposition of extracellular matrix proteins in PCO pathogenesis [4]. The role of cytokines in PCO has been extensively reviewed by Prof. Wormstone [4] and, thus, will not be elaborated further in this article.

### 2.2. Oxidative Stress

In post-cataract surgery, not only is there a significant upregulation of inflammatory and growth factors, but the redox environment also changes drastically. LECs are now in direct contact with the IOL and aqueous humour instead of the outer fibre layer of the lens. The reduced glutathione (GSH) concentration in the outer fibre layer of the lens exceeds 10 mM, while in the aqueous humour, it is only 5 μM [31,32,33]. Following cataract surgery, extracellular GSH concentration in LECs drops sharply. Concurrently, Beebe et al. [34] reported that the partial pressure of oxygen (PO_2_) around the capsular bag increases significantly from 2.8 mmHg in controls to 11.0 mmHg post-surgery. This suggests that LECs are subjected to oxidative stress due to decreased GSH levels and increased oxygen content as they now directly contact the aqueous humour. Oxidative stress continuously assaults LECs due to changes in their microenvironment.

**Figure 2:**
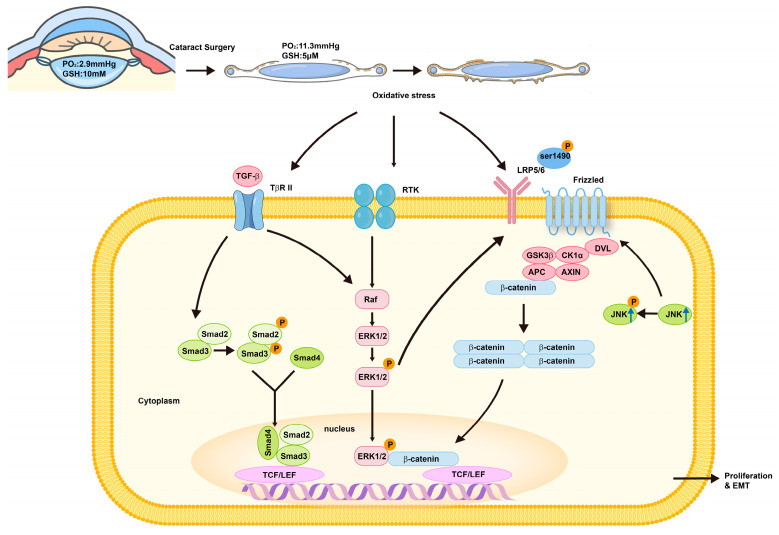
Complex pathological processes during PCO formation under oxidative stress. Lens epithelial cells (LECs) are continuously subjected to oxidative stress due to decreased levels of glutathione (GSH) and increased oxygen content in the aqueous humour following cataract surgery. Both the canonical TGF-β/Smad and Wnt/β-catenin signalling pathways are activated in LEC fibrosis under oxidative stress, contributing to PCO formation. Additionally, the ERK1/2 signalling pathway, which can be activated by TGF-β and oxidative stress, regulates the activation of Wnt/β-catenin, indicating crosstalk between the TGF-β signalling pathway and oxidative stress in PCO formation.

Oxidative stress is known to induce EMT and plays a key role in fibrosis and tumour metastasis [35] in various organs such as the lungs [36,37], liver [38,39], and kidneys [40,41]. Despite this, the relationship between oxidative stress and PCO is under-researched. Yan’s laboratory and others have recently conducted a series of studies exploring this relationship. This article summarises these findings to provide new insights into PCO treatment (Figure 2).

#### 2.2.1. Role of GSH in PCO

To investigate the role of reduced GSH levels in PCO, Fan et al. [32] developed GSH biosynthesis-deficient mice, including lens-conditional gamma glutamyl–cysteine ligase catalytic subunit (Gclc) knockout (KO) mice and systemic gamma glutamyl–cysteine ligase modifier subunit (Gclm) KO mice. Animal experiments demonstrated that reduced GSH levels promote PCO and EMT of LECs. Interestingly, there were no differences in the concentrations of total or active TGF-β2 in the aqueous humour among Gclc KO, Gclm KO, and wild-type (WT) mice, nor were there differences in the phosphorylation of Smad2 and Smad3 in capsular LECs between Gclm KO and WT mice. Transcriptome analysis from the lenses of Gclc KO mice revealed activation of the Wnt/β-catenin signalling pathway, which is directly associated with EMT [42,43]. Then, elevated protein levels of Wnt10a and β-catenin were found in the whole lens and lens epithelium of both Gclc KO and Gclm KO mice compared to WT mice. Importantly, increased EMT marker proteins and active β-catenin nuclear translocation were significantly attenuated by N-acetyl cysteine or GSH ethyl ester treatment after cataract surgery among Gclc KO, Gclm KO, and WT mice.

In human lens, epithelial B3 cells (HLE-B3), and primary porcine LECs, the inhibitor buthionine sulphoximine (BSO) was used to block intracellular GSH biosynthesis, and dimethyl fumarate (DMF) was used to deplete GSH. Cell experiments also demonstrated that reduced GSH levels promoted EMT of LECs, and there were no differences in the phosphorylation of Smad2 and Smad3 in LECs with and without BSO treatment. Following BSO treatment, α-SMA and β-catenin were upregulated in HLE-B3 cells. Importantly, these effects were blocked by XAV939, a Wnt inhibitor. Both animal and cell models confirm that reduced GSH promotes PCO and LEC EMT, with the Wnt/β-catenin pathway being activated rather than the TGF-β pathway.

#### 2.2.2. Oxidative Stress Promotes EMT in LECs

LECs are continuously exposed to oxidative stress, and oxidative stress modulates EMT signalling during fibrosis and metastasis in many organs. It has been confirmed that reduced GSH levels can promote PCO and EMT of LECs via the Wnt/β-catenin signalling pathway [32]. This section focuses on the direct pathogenic role of oxidative stress and the Wnt/β-catenin signalling pathway in the EMT of LECs and PCO formation post-cataract surgery. Yan’s laboratory found that H_2_O_2_, an oxidant, induced the upregulation of EMT marker proteins, proliferation, and migration of primary porcine LECs, indicating that oxidative stress induces EMT in LECs. Additionally, the activation of the TGF-β/Smad signalling pathway, which upregulated the phosphorylation of Smad2/3, and the Wnt/β-catenin signalling pathway, which upregulated β-catenin, Wnt10a, lipoprotein receptor-related protein (LRP6), and phosphorylation of glycogen synthase kinase 3β (GSK-3β) at ser9, were observed during this process. Inhibiting the TGF-β/Smad signalling pathway with LY2109761 or the Wnt/β-catenin signalling pathway with XAV939 partially blocked phenotype and protein marker expression of EMT in LECs, but combining both inhibitors completely blocked it. In mock cataract surgery mice, phosphorylated Smad2 levels in the lens capsule peaked three days post-surgery and then slightly decreased, whereas Wnt10a levels gradually increased until seven days post-surgery. This study demonstrates that oxidative stress promotes EMT of LECs and that both the TGF-β/Smad and Wnt/β-catenin signalling pathways are activated in LEC fibrosis under oxidative stress and PCO formation, with animal experiments indicating that these pathways may play roles at different stages [44].

Other studies corroborate the above findings, highlighting the critical role of oxidative stress in the EMT of LECs. Li et al. [45] found that phosphorylated c-Jun N-terminal kinase 1 (JNK) accumulated in response to H_2_O_2_-induced EMT in LECs. Activated JNK1 removes β-catenin from the cell membrane, causing its nuclear translocation and initiation of transcription. JNK1 siRNA knockdown reverses the effects of H_2_O_2_ on EMT, indicating that JNK1 is necessary for H_2_O_2_-induced EMT in LECs by facilitating β-catenin translocation. Wang et al. [46] explored the relationship between oxidative stress and extracellular vesicles (EVs). EVs derived from a scratch model and H_2_O_2_-treated LECs showed similar increases in the extraction and quantification of EVs. Co-incubation of these EVs with LECs and mouse lenses activated EMT, which was attenuated by reactive oxygen species (ROS) inhibitors. These results suggest that EVs participate in oxidative stress-induced EMT of LECs, making them potential targets for PCO treatment. Above all, oxidative stress plays a key role in the EMT of LECs and PCO formation.

The ERK1/2 signalling pathway is a crucial Smad-independent pathway in TGF-β-induced EMT of LECs. Blocking ERK1/2 signalling with receptor tyrosine kinase (RTK) antagonists effectively inhibits TGF-induced EMT in LECs [47,48,49]. Yan’s laboratory found that the ERK1/2 signalling pathway was activated in LEC fibrosis induced by H_2_O_2_, evidenced by an increased phosphorylated ERK1/2 to total ERK1/2 ratio, which could be reversed by ERK1/2 signalling inhibition. Importantly, ERK1/2 signalling inhibition significantly downregulated the phosphorylation level of LRP6 at ser1490 and GSK-3β at ser9, decreased β-catenin accumulation, and blocked Wnt/β-catenin signalling in LECs under oxidative stress. This indicates a crosstalk between the TGF-β signalling pathway and oxidative stress in PCO formation [50]. TGF-β also stimulates ROS production by downregulating antioxidant genes such as catalase and superoxide dismutase [24]. ROS and TGF-β work together to promote redox imbalance and induce EMT in cancer cells [3,35,51,52]. Thus, investigating the relationship and primary targets between oxidative stress and the TGF-β signalling pathway is crucial for PCO prevention and treatment.

Additionally, GSH plays a vital role in defending against oxidative stress in the cellular redox system [53]. The lens contains several antioxidative defence enzymes besides GSH, which can reverse oxidative damage. Glutaredoxins (Grxs), a specific type of redox-regulating enzyme, work with GSH to mitigate oxidative stress by mediating protein deglutathionylation within the lens. They control redox signalling during growth factor-stimulated cell proliferation and other processes, protecting structural proteins and metabolic enzymes [54]. Yan’s laboratory further focused on Grxs and explored their function in PCO formation.

## 3. Glutaredoxins (Grxs) and PCO

Oxidative stress drives glutathionylation, where free thiol groups on cysteine residues of proteins form protein–glutathione mixed disulfide adducts. Grxs are small thiol proteins that catalyse oxidoreduction reactions. A distinctive feature of Grxs is their ability to catalyse GSH-dependent redox regulation by mediating protein deglutathionylation [55]. Grxs are significantly more efficient than other thioltransferases, such as thioredoxin or protein disulfide isomerase, in catalysing the deglutathionylation step, making the signal reversible [56] and, thus, attracting considerable attention. Grxs play a key role in oxidative signal transduction as deglutathionylation enzymes and are demonstrated by an increasing number of proteins, including protein kinase B (AKT) [57], sirtuin-1 [58], mitofusin [59], and fatty acid-binding protein 5 [60]. These proteins are functionally altered by glutathionylation, and Grxs can reverse these modifications and functional changes.

The two main dithiol Grx isoforms encoded by mammalian cells are nuclear or mitochondrial Grx2 and cytoplasmic Grx1. Both Grx1 [61] and Grx2 [62] were first identified in the lens by Prof. Lou’s laboratory. In the lens, Grx1 is primarily present in the epithelium, showing strong resistance to oxidation, and can protect and repair oxidative stress-induced damage to crystallin proteins and antioxidant enzymes [54]. Grx2 controls mitochondrial redox equilibrium, shielding LECs from oxidative damage and preventing the glutathione-induced inactivation of mitochondrial complex I, which is essential for controlling the electron transport system and ATP synthesis [63,64,65]. Wu et al. [66] reported that the double deletion of Grx1 and Grx2 significantly affected cellular structure and function, leading to aberrant cell cycle progression, disrupted apoptosis, compromised mitochondrial function, and altered cytoskeletal organisation in LECs. These findings highlight the critical roles of Grx1 and Grx2 in preserving cellular redox homeostasis. Both Grx1 and Grx2 play antioxidative roles in LECs, indicating the essential function for maintaining lens transparency.

LECs are directly exposed to the aqueous humour and are continuously affected by oxidative damage after cataract surgery [34]. Yan’s laboratory has investigated the role of Grxs and glutathionylation in the oxidative stress-induced EMT of LECs and the development of PCO.

### 3.1. Role of Grxs in PCO Formation

In a mock cataract surgery mouse model, the protein expression level of Grx1 in the LECs of the lens capsule was significantly downregulated post-surgery compared to the control group. Similarly, in LECs treated with H_2_O_2_, Grx1 expression was also downregulated. Generally, Grx1 deletion leads to an altered cytosolic redox state, elevated levels of S-glutathionylation in proteins, and disruption of redox signalling [55]. In LECs subjected to oxidative stress, Grx1 overexpression reversed these effects, whereas Grx1 knockdown increased S-glutathionylation and reduced the GSH/GSSG ratio. Consistent with Löfgren et al. [67], the loss of Grx1 in LECs increased protein S-glutathionylation, decreased GSH levels, and lowered tolerance to oxidative stress.

Importantly, Yan’s laboratory found that Grx1 overexpression inhibited the proliferation, migration, and expression of EMT marker proteins in LECs exposed to H_2_O_2_. Yan’s laboratory constructed Grx1 systemic KO mice via CRISPR/Cas-mediated genome engineering and performed extracapsular lens extraction on both Grx1 KO and WT mice. The migration and proliferation of capsular LECs were more severe in Grx1 KO mice than in WT mice. Using both in vivo and in vitro models, it demonstrated that Grx1 regulated the S-glutathionylation of proteins and the GSH/GSSG ratio in PCO formation and EMT of LECs [68].

Grx2, a key antioxidant enzyme in the mitochondria, regulates primary ROS signalling via S-glutathionylation of mitochondrial proteins [69,70]. In both the mock cataract surgery model and H_2_O_2_-treated LECs, Yan’s laboratory observed that Grx2 protein expression was decreased compared to controls. Additionally, a regulatory role for Grx2 in mitochondrial ROS (mROS) and overall ROS levels in LECs under oxidative stress were identified. Loss of Grx2 exacerbated ROS and mROS generation in LECs under oxidative stress, whereas Grx2 overexpression protected LECs from ROS and mROS damage. It is well-established that ROS and mROS are crucial in controlling cell proliferation, activating oncogenes, migratory signalling, and LEC EMT [46,70].

The investigations of Yan’s laboratory revealed that Grx2 overexpression shielded LECs from EMT, while deletion of Grx2 enhanced migration, proliferation, and expression of EMT marker proteins in LECs under oxidative stress. Yan’s laboratory developed Grx2 systemic KO and knock-in (KI) mice via CRISPR/Cas-mediated genome engineering. Five days after mock surgery, the Grx2 KO mouse model exhibited a greater number of proliferating and migrating LECs in the anterior and posterior capsules compared to the WT model; this proliferation was reduced in the Grx2 KI model. The findings suggest that Grx2 KI ameliorates PCO and EMT in LECs, likely due to reduced mROS and ROS levels and improved Grx2-mediated oxidative defence [71].

### 3.2. Mechanism of Grxs in PCO Formation

After confirming the importance of Grxs in PCO formation, we further reviewed its mechanism. Casein kinase 1α (CK1α), identified by liquid chromatography–tandem mass spectrometry (LC-MS/MS) in LECs under oxidative stress, is a critical suppressor of the Wnt/β-catenin signalling cascade [72]. Reduced Grx1 promotes the EMT of LECs and exacerbates PCO by upregulating glutathionylation at cysteine 249 of CK1α. This glutathionylation inhibits CK1α kinase activity, specifically the phosphorylation of β-catenin at ser45, thereby promoting nuclear translocation of β-catenin and activating the Wnt/β-catenin signalling pathway. Therefore, targeting Grx1 and CK1α deglutathionylation could be promising therapeutic approaches for PCO [68].

The Wnt/β-catenin signalling pathway plays a crucial role in many diseases, particularly cancer [73,74,75,76,77,78], where CK1α functions as a negative regulator, making it a desirable target for medical intervention. Further studies should explore Grx1 and CK1α glutathionylation in diseases related to the Wnt/β-catenin signalling pathway, with a focus on understanding the fundamental processes that lead to CK1α activation or inactivation. This could provide a foundation for developing effective treatments.

To investigate the mechanism of Grx2 in PCO formation, Yan’s laboratory analysed the lens capsules of Grx2 KO and WT mice after mock surgery using LC-MS/MS. The analysis revealed that the expression of integrin-linked kinase (ILK), a protein located in focal adhesion complexes, was significantly increased in the Grx2 KO group compared to the WT group. ILK is essential for cell migration, proliferation, and adhesion [79]. Both in vivo and in vitro models demonstrated that Grx2 protected against PCO formation by regulating ILK expression, and Grx2 deficiency-induced elevated ILK levels, which phosphorylated AKT and GSK-3β, stimulating the Wnt/β-catenin pathway and promoting PCO development and EMT in LECs. These findings suggest a potential strategy for preventing PCO.

**Figure 3:**
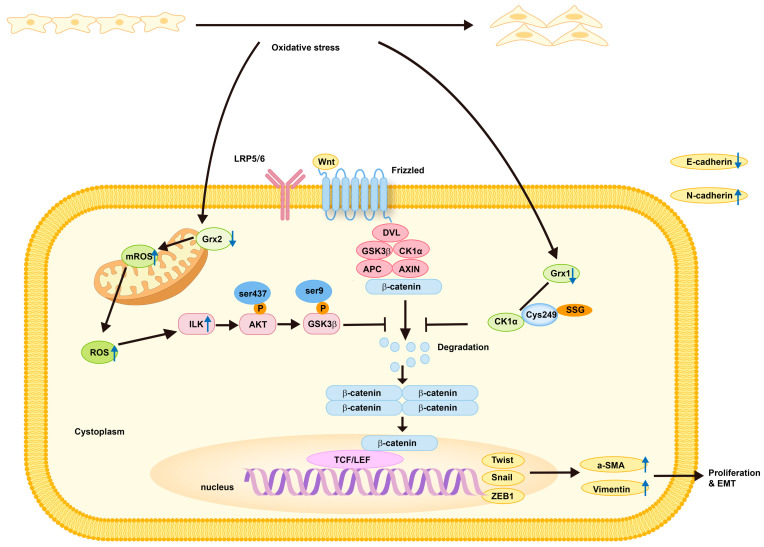
Role and mechanism of Grx1 and Grx2 in protection against PCO formation. Reduced Grx1, present in PCO formation and LECs under oxidative stress, promotes EMT of LECs and aggravates PCO by upregulating glutathionylation at cysteine 249 of CK1α. This upregulation blocks degradation of β-catenin, enhances the nuclear translocation of β-catenin, and activates the Wnt/β-catenin signalling pathway. Additionally, increased ILK, induced by Grx2 deficiency, phosphorylates AKT and GSK-3β, thereby further blocking degradation of β-catenin, enhancing the nuclear translocation of β-catenin, and activating the Wnt/β-catenin signalling pathway, which promotes PCO formation and EMT in LECs.

Grx2 may inhibit oxidative stress-induced ILK elevation by suppressing cytoplasmic ROS production and stabilising mROS levels through the deglutathionylation of mitochondrial proteins. However, the specific mechanism requires further investigation [71]. Interestingly, a recent study indicated that ILK could affect mitochondrial reprogramming and trafficking [80], suggesting that ILK might not only be regulated by Grx2 but also jointly influence mitochondrial function and regulate oxidative stress-related cell migration with Grx2 (Figure 3).

## 4. Advanced Antioxidative Pharmacological Agents

Research from Yan’s laboratory and other scientists has established that oxidative stress was a critical factor in PCO formation, and Grxs could serve as therapeutic targets. Exogenous administration of recombinant Grx1 has been shown to halt the increase in protein S-glutathionylation (PSSG) induced by lung fibrosis [81], while recombinant Grx2 has been effective in preventing airway inflammation [82], demonstrating the therapeutic potential of recombinant Grxs. For PCO treatment, administration methods, dosages, and forms are worthy of further exploration and require multidisciplinary collaboration [68]. Additionally, the redox state of cells, particularly the GSH/GSSG ratio, appears to be crucial for Grx1 activation [55], suggesting that increasing GSH levels could be a promising direction for PCO treatment. Maddirala et al. [83] reported that N-acetylcysteine (NAC) eye drops increased GSH levels in a rat model and prevented selenite-induced cataracts. However, the impact of NAC eye drops on PCO and the appropriate dosing need further exploration. The development of efficient transepithelial and long-acting NAC eye drops is essential for treating diseases related to oxidative damage [84].

Nanotechnology-based ocular drug delivery systems, including IOLs, hydrogels, and nanoparticles, hold the potential for overcoming delivery barriers [5,85]. Rong et al. [86] proposed a heterobifunctional adaptor (2-formylbenzeneboronic acid) for assembling cargo proteins and phenolic polymers into stable nanoparticles for direct delivery of the antioxidant proteins. In an animal model, superoxide dismutase was successfully injected intravitreally, significantly increasing glutathione reductase activity, reducing retinal malondialdehyde levels, and ameliorating oxidative stress and cellular damage induced by ischemia–reperfusion with minimal adverse effects. Intracellular delivery systems are expected to bring breakthroughs in treating oxidative stress-related eye diseases and may facilitate Grxs delivery for PCO treatment.

Zhang et al. [87] developed dual dexamethasone (Dex)- and curcumin (Cur)-loaded ROS-responsive nanoparticles (CPDC NPs) with anti-inflammatory and antioxidative effects, which demonstrated superior therapeutic outcomes compared to commercial eye drops in an endotoxin-induced uveitis rabbit model. These CPDC NPs not only efficiently released dexamethasone and curcumin in oxidative physiological environments but were also effectively incorporated into activated macrophages, suppressing proinflammatory factors. Considering the significance of inflammation and oxidative stress in PCO pathogenesis, dual-drug-loaded ROS-responsive NPs offer a promising solution and have substantial application potential.

Post-cataract surgery, an IOL is implanted into the lens capsule. Using IOL as a medium for PCO prophylaxis is a direct and effective approach that has shown promise in various clinical applications. Research focuses on optimising IOL materials, designs, and IOL surface modifications, such as chemical grafting, drug loading, plasma, ultraviolet, and ozone treatments, coating modifications, and layer-by-layer self-assembly techniques [88]. Novel IOLs, including Dox@Exos-IOLs [89], INDOM-IOL [90], and AuNPs@MIL-PGE bulk materials [91], have demonstrated effectiveness in animal models; however, their clinical application lacks sufficient evidence. There is a notable gap in developing novel anti-inflammatory and antioxidative IOLs, which could offer a promising alternative for clinical application in PCO prevention and warrant further investigation.

## 5. Conclusions and Future Directions

In summary, we have reviewed the epidemiology, clinical manifestations, and pathogenesis of PCO, with a particular emphasis on the role of oxidative stress in its development. We have also highlighted the protective roles and mechanisms of Grx1 and Grx2 against PCO formation, underscoring their potential as targets for drug treatment. Additionally, there has been significant progress in the development of PCO drugs and IOLs. Given the critical role of oxidative stress in PCO, we advocate for the development of drugs and IOLs that target both antioxidant and anti-inflammatory pathways. This dual approach may offer more effective prevention and treatment strategies for PCO. At the same time, the safety and stability of intraocular drugs or materials should be rigorously tested and evaluated.
